# Associations of smoking, body mass index, dietary lutein, and the *LIPC* gene variant rs10468017 with advanced age-related macular degeneration

**Published:** 2010-11-17

**Authors:** Johanna M. Seddon, Robyn Reynolds, Bernard Rosner

**Affiliations:** 1 Ophthalmic Epidemiology and Genetics Service, Department of Ophthalmology, Tufts Medical Center, Boston, MA; 2 Tufts University School of Medicine, Boston, MA; 3 Channing Laboratory, Brigham and Women’s Hospital, Boston, MA

## Abstract

**Objective:**

A novel locus in the hepatic lipase (*LIPC*) gene was found to be significantly related to advanced age-related macular degeneration (AMD) in our genome-wide association study. We evaluated its association and interaction with previously identified genetic variants and modifiable factors.

**Methods:**

Participants in the Age-Related Eye Disease Study with advanced AMD (n=545 cases) or no AMD (n=275 controls) were evaluated. AMD status was determined using fundus photography. Covariates included cigarette smoking, body mass index (BMI), and dietary lutein. Individuals were genotyped for the rs10468017 polymorphism in *LIPC* as well as seven previously identified AMD genetic loci. Unconditional logistic regression analyses were then performed.

**Results:**

The TT genotype of the *LIPC* variant was associated with a reduced risk of AMD, with odds ratios (OR) of 0.50 (95% confidence interval (CI) 0.20–0.90) and p=0.014 for the TT genotype versus the CC genotype, controlling for age, gender, smoking, body mass index (BMI), and nutritional factors. Controlling for seven other AMD genetic variants, the OR was 0.50, 95% (CI 0.20–1.1, p=0.077). The magnitude of the effect was similar for both atrophic and neovascular forms of AMD. Cigarette smoking and higher BMI increased the risk, while higher dietary lutein reduced the risk of advanced AMD, adjusting for genetic variants. There were no significant interactions between *LIPC* and smoking, BMI, or lutein. There was a possible association between *LIPC* and complement factor H (*CFH)* rs1410996, and a possible interaction effect between *LIPC* and both *CFH* rs10033900 and the complement factor I (*CFI)* variants in terms of risk of AMD.

**Conclusions:**

*LIPC* is associated with reduced risk of advanced AMD, independent of demographic and environmental variables. Both genetic susceptibility and behavioral and lifestyle factors modify the risk of developing AMD.

## Introduction

The links between genetics, environment and age-related macular degeneration (AMD) have been assessed in several previous studies. The US twin study of AMD quantified the proportions of variance in early, intermediate, and advanced forms of this disease due to genetic and environmental factors as 46%–71% and 19%–37%, respectively [1,2]. Several environmental factors have been identified, including cigarette smoking [3,4], higher body mass index (BMI) [5,6], and dietary carotenoids [7–10]. A genetic effect was suggested for several years based on clinical observations, familial aggregation and linkage studies [1,2,11–15], and has been confirmed by studies showing associations between AMD and several genetic loci [16–30]. These genetic loci are estimated to account for approximately one-half of the heritability of AMD [22].

In an attempt to identify other susceptibility loci and to explain the remaining heritability of AMD, we conducted a large genome-wide association study (GWAS) of 979 cases of advanced AMD and 1709 controls, with replication of our top results in independent cohorts with a total of 5789 cases and 4234 controls [29]. Our scan identified the hepatic lipase gene (*LIPC*) in the high-density lipoprotein cholesterol (HDL) pathway as a novel locus for AMD risk, with a protective effect for the minor T allele. A separate GWAS corroborated the *LIPC* association with AMD [30]. *LIPC* encodes hepatic triglyceride lipase, which is expressed in the liver. One of the principal functions of the enzyme hepatic lipase is to convert HDL to LDL. *LIPC* performs the dual functions of triglyceride hydrolase and ligand bridging factors for receptor-mediated lipoprotein uptake [29]. We further explored this *LIPC* locus and found that the association was strongest at the functional variant in the promoter region (single nucleotide polymorphism (SNP) rs10468017), which influences *LIPC* expression [29].

In this report, we expanded upon the results of the GWAS discovery of the *LIPC* gene by evaluating the association between the *LIPC* genetic variant and other genes related to advanced AMD, exploring the relationship between this gene and the two distinct advanced “dry and wet” phenotypes, and assessing *LIPC* gene-environment associations and interactions with demographic, personal and lifestyle factors.

## Methods

The Age-Related Eye Disease Study (AREDS) included a randomized clinical trial to assess the effect of antioxidant and mineral supplements on risk of AMD and cataract as well as a longitudinal study of progression of AMD that ended in December, 2005 [8]. Based on ocular examination and reading center photographic grading of fundus photographs, participants with European ancestry in this study were divided into two main groups representing the most discordant phenotypes: no AMD defined as either no drusen or non-extensive small drusen (n=275), or advanced AMD with visual loss (n=545). The advanced form of AMD, which is associated with visual loss, was then reclassified into the two subtypes of either non-central or central geographic atrophy (GA, n=139) or neovascular disease (NV, n=406), independent of visual acuity level, using the Clinical Age-Related Maculopathy Grading System [31], to determine whether results differed between the two advanced AMD phenotypes. Ethnicity and risk factor data were obtained at the baseline visit from questionnaires as well as measurements of height and weight.

All genotyping was performed using primer mass extension and MALDI-TOF MS analysis according to the MassEXTEND methodology of Sequenom (San Diego, CA) at the Broad Institute Center for Genotyping and Analysis, Cambridge, MA [32]. The single nucleotide polymorphism (SNP), rs10468017, which is a functional variant of the *LIPC* gene on chromosome 15q22, was assessed. In addition, variants in seven other known AMD genes were also determined: 1) the common SNP in exon 9 of the complement factor H (*CFH)* gene on chromosome 1q31 (rs1061170), a change 1277T>C, resulting in a substitution of histidine for tyrosine at codon 402 of the CFH protein, Y402H; 2) *CFH* rs1410996, an independently associated SNP variant within intron 14 of *CFH*; 3) SNP rs10490924 in the *ARMS2/HTRA1* region of chromosome 10, a non-synonymous coding SNP variant in exon 1, resulting in a substitution of the amino acid serine for alanine at codon 69; 4) Complement component 2 or *C2 E318D* (rs9332739), the non-synonymous coding SNP variant in exon 7 of *C2* resulting in the amino acid glutamic acid changing to aspartic acid at codon 318; 5) Complement Factor B or *CFB R32Q* (rs641153), the non-synonymous coding SNP variant in exon 2 of *CFB*, resulting in the amino acid glutamine changing to arginine at codon 32; 6) Complement component 3 or *C3 R102G* (rs2230199), the non-synonymous coding SNP variant in exon 3 of *C3*, resulting in the amino acid glycine to arginine at codon 102 on chromosome 19; and 7) Complement Factor I or *CFI* (rs10033900) on chromosome 4. The genetic variant on chromosome 10, *ARMS2/HTRA1*, remains a subject of debate as to whether the gene *HTRA1* adjacent to it may in fact be the AMD-susceptibility gene on 10q26 [26,27]; however, the relevant SNPs in these two genes have been reported to be nearly perfectly correlated. Thus, while the other SNP is a promising candidate variant, rs10490924 used in this study can be considered a surrogate for the causal variant that resides in this region. For the *C2/CFB* genes, there are two independent associations to the *C2/CFB* locus, but because of linkage disequilibrium, we do not know which of the two genes or if in fact both are functionally affected.

### Statistical analyses

Logistic regression was used to determine the association between *LIPC* genotypes and other risk factors. Individuals with advanced AMD, as well as the GA and NV subtypes, were compared to the control group of persons with no AMD in regards to genotype and risk factor data. Multivariate unconditional logistic regression analyses were performed to evaluate the relationships between AMD and *LIPC*, controlling for age (70 or older, younger than 70); gender; education (high school or less, more than high school); cigarette smoking (never, past, current); BMI, which was calculated as the weight in kilograms divided by the square of the height in meters (<25, 25–29.9, and ≥30); dietary lutein (micrograms), which was determined from food frequency questionnaires, divided into tertiles, and adjusted for sex and calorie intake because men tend to have a higher calorie intake than women; and assignment to a supplement containing antioxidants or a supplement not containing antioxidants. We included dietary lutein in our models because it is related to AMD [7,10,33] and because HDL is the major lipoprotein transporter of lutein and zeaxanthin in the body; moreover, the T allele of the *LIPC* gene increases HDL [29,34–36].

A separate statistical model including all of the above factors, plus the seven other genetic variants, was also evaluated. The association between the *LIPC* gene and these variants was assessed. Tests for multiplicative interactions between genes and between genes and environmental factors were performed using cross-product terms according to genotype and the individual risk factors [37]. Odds ratios and 95% confidence intervals were calculated for each risk factor and within the genotype groups.

## Results

The distributions of demographic, personal, and lifestyle variables, previously shown to be associated with AMD in studies of this cohort [6,33,38,39], are shown in Table 1 according to the *LIPC* genotypes for controls and cases with geographic atrophy and neovascular disease. There were no significant differences in gender, education, smoking, BMI, antioxidant supplements, or calorie-adjusted dietary lutein among the *LIPC* genotypes. There was a significant association between the number of T alleles and age for the NV group, which was not seen in the other groups.

**Table 1:** Distribution of demographic and behavioral risk factors for advanced age-related macular degeneration and controls according to hepatic lipase-C (*LIPC*) genotypes.

** **	**Controls**							**Geographic atrophy**						
** **	**CC**		**CT**		**TT**		** **	**CC**		**CT**		**TT**		** **
***LIPC*: rs10468017 Genotype**	**N**	**%**	**N**	**%**	**N**	**%**	p value*	**N**	**%**	**N**	**%**	**N**	**%**	p value*
**Baseline age**														
50-69	89	(70)	92	(76)	21	(81)		38	(46)	28	(56)	4	(57)	
70-95	39	(30)	29	(24)	5	(19)	0.30	44	(54)	22	(44)	3	(43)	0.57
**Gender**														
Male	58	(45)	57	(47)	10	(38)		44	(54)	24	(48)	3	(43)	
Female	70	(55)	64	(53)	16	(62)	0.49	38	(46)	26	(52)	4	(57)	0.58
**Education**														
High School or Less	7	(5)	4	(3)	1	(4)		7	(9)	6	(12)	0	0.0	
College or more	121	(95)	117	(97)	25	(96)	0.80	75	(91)	44	(88)	7	100.0	0.47
**Smoking **														
Never	67	(52)	56	(46)	15	(58)		30	(37)	16	(32)	3	(43)	
Former	58	(45)	57	(47)	10	(38)	0.50	42	(51)	31	(62)	3	(43)	0.71
Current	3	(2)	8	(7)	1	(4)	0.99	10	(12)	3	(6)	1	(14)	1.00
**BMI **														
<25.0	39	(30)	40	(33)	9	(35)		23	(28)	16	(32)	3	(43)	
25.0-29.9	55	(43)	59	(49)	13	(50)	0.98	35	(43)	20	(40)	1	(14)	0.17
≥ 30.0	34	(27)	22	(18)	4	(15)	0.35	24	(29)	14	(28)	3	(43)	0.97
P for Trend							0.40							0.97
**Supplements**														
No Antioxidants	69	(54)	66	(55)	17	(65)		44	(54)	30	(60)	4	(57)	
Antioxidants	59	(46)	55	(45)	9	(35)	0.28	38	(46)	20	(40)	3	(43)	0.84
**Lutein: Calorie Adjusted, Sex specific (mean µg)**														
Males		1543.1		1703.1		1520.6	0.55		1329.5		1549.6		1100.7	0.67
Females		1468.0		1374.1		1607.8	0.91		1221.2		1361.6		1193.4	0.65
** **	**Neovascular disease**							**Combined advanced AMD**						
** **	**CC**		**CT**		**TT**		** **	**CC**		**CT**		**TT**		** **
***LIPC*: rs10468017 Genotype**	**N**	**%**	**N**	**%**	**N**	**%**	p value*	**N**	**%**	**N**	**%**	**N**	**%**	p value*
**Baseline Age**														
50-69	102	(48)	89	(51)	4	(19)	** **	140	(48)	117	(52)	8	(29)	** **
70-95	109	(52)	85	(49)	17	(81)	0.01	153	(52)	107	(48)	20	(71)	0.05
**Gender**														
Male	88	(42)	64	(37)	6	(29)	** **	132	(45)	88	(39)	9	(32)	** **
Female	123	(58)	110	(63)	15	(71)	0.24	161	(55)	136	(61)	19	(68)	0.18
**Education**														
High School or Less	32	(15)	11	(6)	5	(24)	** **	39	(13)	17	(8)	5	(18)	** **
College or more	179	(85)	163	(94)	16	(76)	0.24	254	(87)	207	(92)	23	(82)	0.48
**Smoking **														
Never	78	(37)	70	(40)	11	(52)	** **	108	(37)	86	(38)	14	(50)	** **
Former	108	(51)	82	(47)	8	(38)	0.18	150	(51)	113	(50)	11	(39)	0.17
Current	25	(12)	22	(13)	2	(10)	0.48	35	(12)	25	(11)	3	(11)	0.53
**BMI **														
<25.0	45	(21)	51	(29)	4	(19)	** **	68	(23)	67	(30)	7	(25)	** **
25.0-29.9	100	(47)	74	(43)	10	(48)	0.86	135	(46)	94	(42)	11	(39)	0.63
≥ 30.0	66	(31)	49	(28)	7	(33)	0.79	90	(31)	63	(28)	10	(36)	0.87
p for Trend	** **	** **	** **	** **	** **	** **	0.80	** **	** **	** **	** **	** **	** **	0.84
**Supplements**														
No Antioxidants	109	(52)	84	(48)	8	(38)	** **	153	(52)	114	(51)	12	(43)	** **
Antioxidants	102	(48)	90	(52)	13	(62)	0.24	140	(48)	110	(49)	16	(57)	0.34
**Lutein: Calorie Adjusted, Sex specific (mean µg)**														
Males	** **	1430.4	** **	1651.6	** **	1295.3	0.28	** **	1395.9	** **	1623.1	** **	1226.9	0.25
Females	** **	1254.8	** **	1203.0	** **	1315.6	0.86	** **	1246.8	** **	1231.8	** **	1288.9	0.98

*p value indicates the relationship between the number of T alleles for *LIPC* and each category of the variable.

The associations between *LIPC* and other known AMD genetic loci are shown in Table 2. There was a possible association between *LIPC* and CFH rs1410996 in the GA subgroup (p=0.035). There were no other significant associations between the *LIPC* gene and other AMD genetic loci among the controls or in the advanced AMD phenotypes.

**Table 2:** Associations between hepatic lipase-C (*LIPC*) genotypes and ather AMD related genetic variants

** **	**Controls**							**Geographic atrophy**						
** **	**CC**		**CT**		**TT**			**CC**		**CT**		**TT**		
***LIPC*: rs10468017 Genotype**	**N**	**%**	**N**	**%**	**N**	**%**	p value*	**N**	**%**	**N**	**%**	**N**	**%**	p value*
***CFH*: rs1061170 (Y402H)**														
TT	42	(33)	57	(47)	14	(54)		14	(17)	8	(16)	1	(14)	
CT	67	(52)	48	(40)	8	(31)		32	(39)	20	(40)	3	(43)	
CC	19	(15)	16	(13)	4	(15)	0.096	36	(44)	22	(44)	3	(43)	0.86
***CFH*: rs1410996**														
TT	16	(13)	25	(21)	6	(23)		1	(1)	2	(4)	1	(14)	
CT	65	(51)	64	(53)	14	(54)		27	(33)	11	(22)	3	(43)	
CC	47	(37)	32	(27)	6	(23)	0.23	54	(66)	37	(74)	3	(43)	0.035
***ARMS2*/*HTRA1*: rs10490924**														
GG	89	(70)	78	(65)	16	(62)		27	(33)	21	(42)	3	(43)	
GT	34	(28)	39	(32)	10	(39)		40	(49)	24	(48)	4	(57)	
TT	3	(2)	4	(3)	0	0.0	0.60	15	(18)	5	(10)	0	0.0	0.18
***CFB*: rs641153 (R32Q)**														
CC	101	(79)	91	(75)	19	(73)		76	(93)	47	(94)	7	100.0	
CT/TT	27	(21)	30	(25)	7	(27)	0.57	6	(7)	3	(6)	0	0.0	0.45
***C2*: rs9332739 (E318D)**														
GG	116	(91)	106	(88)	24	(92)		79	(96)	49	(98)	7	100.0	
CG/CC	12	(9)	15	(12)	2	(8)	0.71	3	(4)	1	(2)	0	0.0	0.57
***C3*: rs2230199 (R102H)**														
CC	82	(64)	74	(61)	14	(54)		42	(51)	25	(50)	4	(57)	
CG	44	(34)	40	(33)	11	(42)		33	(40)	23	(46)	2	(29)	
GG	2	(2)	7	(6)	1	(4)	0.60	7	(9)	2	(4)	1	(14)	0.66
***CFI*: rs10033900**														
CC	36	(28)	40	(33)	11	(42)		18	(22)	7	(14)	1	(14)	
CT	59	(46)	64	(53)	11	(42)		40	(49)	28	(56)	4	(57)	
TT	33	(26)	17	(14)	4	(15)	0.30	24	(29)	15	(30)	2	(29)	0.99
	**Neovascular AMD**							**Combined Advanced AMD**						
***LIPC*: rs10468017 genotype**	**CC**		**CT**		**TT**			**CC**		**CT**		**TT**		
	**N**	**%**	**N**	**%**	**N**	**%**	p value*	**N**	**%**	**N**	**%**	**N**	**%**	p value*
***CFH*: rs1061170 (Y402H)**														
TT	37	(18)	24	(14)	4	(19)		51	(17)	32	(14)	5	(18)	
CT	98	(47)	77	(44)	9	(43)		130	(44)	97	(43)	12	(43)	
CC	76	(36)	73	(42)	8	(38)	0.88	112	(38)	95	(42)	11	(39)	0.10
***CFH*: rs1410996**														
TT	10	(5)	5	(3)	1	(5)		11	(4)	7	(3)	2	(7)	
CT	62	(29)	44	(25)	6	(29)		89	(30)	55	(25)	9	(32)	
CC	139	(66)	125	(72)	14	(67)	0.99	193	(66)	162	(72)	17	(61)	0.34
***ARMS2*/*HTRA1*rs10490924**														
GG	62	(29)	48	(28)	6	(29)		89	(30)	69	(31)	9	(32)	
GT	108	(51)	83	(48)	8	(38)		148	(51)	107	(48)	12	(43)	
TT	41	(19)	43	(25)	7	(33)	0.16	56	(19)	48	(21)	7	(25)	0.49
***CFB*: rs641153 (R32Q)**														
CC	191	(90)	162	(93)	20	(95)		267	(91)	209	(93)	27	(96)	
CT/TT	20	(10)	12	(7)	1	(5)	0.45	26	(9)	15	(7)	1	(4)	0.31
***C2*: rs9332739 (E318D)**														
GG	197	(93)	167	(96)	21	100		276	(94)	216	(96)	28	100.0	
CG/CC	14	(7)	7	(4)	0	0.0	0.19	17	(6)	8	(4)	0	0.0	0.16
***C3*: rs2230199 (R102H)**														
CC	98	(47)	88	(51)	10	(48)		140	(48)	113	(50)	14	(50)	
CG	95	(45)	72	(41)	10	(48)		128	(44)	95	(42)	12	(43)	
GG	18	(9)	14	(8)	1	(5)	0.56	25	(9)	16	(7)	2	(7)	0.77
***CFI*: rs10033900**														
CC	47	(22)	41	(24)	6	(29)		65	(22)	48	(21)	7	(25)	
CT	118	(56)	76	(44)	12	(57)		158	(54)	104	(46)	16	(57)	
TT	46	(22)	57	(33)	3	(14)	0.44	70	(24)	72	(32)	5	(18)	0.50

*P for trend for overall association between number of T alleles for *LIPC* and number of risk/protective alleles for other genotypes, or presence of at least 1 risk/protective allele for *CFB* and *C2*.

Table 3 shows the odds ratios based on the multivariate models, comparing all advanced AMD cases, as well as GA and NV cases, with controls for the *LIPC* variant, while adjusting for demographic and behavioral risk factors. Controlling for age, gender, education, smoking, BMI, AREDS treatment, and dietary lutein in multivariate model 1 (MV1), the OR was 0.5 (95% confidence interval (CI) 0.2–0.9) comparing the TT genotype to the CC genotype for advanced AMD (p=0.014), which suggests a protective effect for the TT genotype. Controlling for the other seven genotypes (multivariate model 2), did not alter the magnitude of the effect of this new genetic variant (OR 0.5, 95% CI 0.2–1.1), although this was not statistically significant possibly due to small numbers. There were minimal differences between GA and NV for this locus. For GA in model 1, the OR was 0.5 (95% CI 0.2–1.3) for the TT genotype, and for NV, the OR was 0.4 (95% CI 0.2–0.9).

**Table 3:** Multivariate analyses of associations between advanced age-related macular degeneration (AMD), hepatic lipase-C (*LIPC*) genotypes, and demographic, genetic, and behavioral risk factors.

** **	**Combined advanced AMD**		**Geographic atrophy**		**Neovascular AMD**	
** **	**545/275**		**139/275**		**406/275**	
**Number of cases/controls**	**OR (CI)**	**p value**	**OR (CI)**	**p value**	**OR (CI)**	**p value**
***LIPC* genotype**						
Multivariate model 1						
CC	1.0		1.0		1.0	
CT	0.9 (0.7-1.2)	0.529	0.7 (0.4-1.1)	0.143	1.0 (0.7-1.4)	0.87
TT	0.5 (0.2-0.9)	0.014	0.5 (0.2-1.3)	0.152	0.4 (0.2-0.9)	0.02
Number of T alleles (p - trend)		0.047		0.062		0.105
Multivariate Model 2						
CC	1.0		1.0		1.0	
CT	1.0 (0.6 - 1.4)	0.846	0.9 (0.5-1.6)	0.805	1.0 (0.6-1.5)	0.97
TT	0.5 (0.2 - 1.1)	0.077	0.7 (0.2-2.2)	0.555	0.4 (0.2-1.1)	0.066
Number of T alleles (p - trend)		0.21		0.587		0.22
**Age**						
Multivariate Model 1						
<70	1.0		1.0		1.0	
≥70	3.2 (2.3 - 4.4)	<0.0001	2.9 (1.8 - 4.6)	<0.0001	3.3 (2.3 - 4.7)	<0.0001
Multivariate Model 2						
<70	1.0		1.0		1.0	
≥70	3.3 (2.2 - 4.9)	<0.0001	3.1 (1.8 - 5.4)	<0.0001	3.9 (2.5 - 6.0)	<0.0001
**Gender**						
Multivariate Model 1						
Male	1.0		1.0		1.0	
Female	0.8 (0.6 - 1.1)	0.125	1.2 (0.8 - 1.9)	0.437	0.7 (0.5 - 1.0)	0.026
Multivariate Model 2						
Male	1.0		1.0		1.0	
Female	0.9 (0.6 - 1.3)	0.42	1.2 (0.7 - 2.1)	0.442	0.7 (0.5 - 1.1)	0.13
**Education**						
Multivariate Model 1						
≤HS	1.0		1.0		1.0	
>HS	0.4 (0.2 - 0.8)	0.007	0.4 (0.2 - 1.0)	0.061	0.4 (0.2 - 0.7)	0.005
Multivariate Model 2						
≤HS	1.0		1.0		1.0	
>HS	0.5 (0.2 - 1.1)	0.079	0.4 (0.1 - 1.1)	0.074	0.6 (0.2 - 1.4)	0.2
**Smoking**						
Multivariate Model 1						
Ever	1.0		1.0		1.0	
Past	1.5 (1.1 - 2.2)	0.01	1.8 (1.1 - 2.9)	0.016	1.5 (1.1 - 2.1)	0.024
Current	3.9 (2.0 - 7.7)	<0.0001	4.0 (1.6 - 9.6)	0.002	3.9 (1.9 - 7.9)	0.0002
Multivariate Model 2						
Never	1.0		1.0		1.0	
Past	2.0 (1.3 - 3.0)	0.001	1.8 (1.0 - 3.1)	0.054	2.1 (1.3 - 3.3)	0.001
Current	4.5 (2.1 - 10.1)	0.0002	4.3 (1.4 - 13.0)	0.009	4.8 (2.1 - 11.2)	0.0002
**BMI**						
Multivariate Model 1						
<25	1.0		1.0		1.0	
25 - 29.9	1.3 (0.9 - 1.9)	0.15	1.1 (0.6 - 1.8)	0.85	1.4 (1.0 - 2.1)	0.085
>=30	2.0 (1.3 - 3.1)	0.002	1.8 (1.0 - 3.2)	0.057	2.1 (1.3 - 3.4)	0.002
(p-trend)		0.002		0.064		0.002
Multivariate Model 2						
<25	1.0		1.0		1.0	
25 - 29.9	1.2 (0.7 - 1.9)	0.5	0.9 (0.5 - 1.7)	0.72	1.4 (0.8 - 2.3)	0.20
>=30	2.0 (1.2 - 3.3)	0.01	1.7 (0.8 - 3.5)	0.15	2.3 (1.3 - 4.0)	0.005
(p-trend)		0.01		0.81		0.005
**Calorie adjusted, sex-specific Lutein (tertiles)**						
Multivariate Model 1						
1st tertile	1.0		1.0		1.0	
2nd tertile	0.7 (0.5 - 1.1)	0.13	0.7 (0.4 - 1.3)	0.25	0.8 (0.5 - 1.2)	0.27
3rd tertile	0.6 (0.4 - 1.0)	0.029	0.6 (0.4 - 1.1)	0.11	0.7 (0.4 - 1.0)	0.056
(p - trend)		0.031		0.114		0.057
Multivariate Model 2						
1st tertile	1.0		1.0		1.0	
2nd tertile	0.8 (0.5 - 1.3)	0.46	0.7 (0.4 - 1.3)	0.28	0.9 (0.6 - 1.6)	0.79
3rd tertile	0.7 (0.4 - 1.1)	0.099	0.6 (0.3 - 1.1)	0.077	0.8 (0.5 - 1.3)	0.30
(p - trend)		0.097		0.078		0.29

OR=Odds Ratio, CI=95% Confidence Interval Multivariate 1=Model Adjusted for age (50 - 69, 70 −95), gender, education (≤ high school versus > high school), smoking (never, past, current), BMI (<25, 25 - 29.9, ≥30), *LIPC* (CC, CT, TT), antioxidant treatment (supplement containing antioxidants versus supplement containing no antioxidants), calorie adjusted lutein (tertile ranges micrograms: females-1=170.7 - 1096.6, 2=1096.7 - 1685.8, 3=1685.9 - 7259.0; males-1=151.4 - 1107.7, 2=1107.8 - 1669.0, 3=1669.1 - 11614.0) Multivariate 2=Model Adjusted for all variables in Multivariate 1 plus *CFH* Y402H (TT, CT, CC), *CFH* rs1410966 (TT, CT, CC), *C2* (GG, CG/CC), *CFB* (CC,CT/TT), *ARMS2*/*HTRA1* (GG, GT, TT), *C3* (CC, CG, GG), *CFI* (CC, CT, TT).

Table 3 also shows the associations between advanced AMD, GA, and NV with older age, less education, cigarette smoking (past and current), higher BMI, and lower levels of dietary lutein intake, compared with controls and controlling for the *LIPC* genotype. Cigarette smoking was associated with a statistically significant increased risk of advanced AMD for both subtypes, controlling for genotype and other factors. ORs in the multivariate model 1 (demographic, environmental factors and *LIPC* genetic variant) range from 3.9 to 4.0 for current smoking and 1.5–1.8 for past smoking. A body mass index of 30 kg/m^2^ or higher increased the risk for advanced AMD for both neovascular cases (OR 2.1, 95% CI 1.3–3.4) and for geographic atrophy (OR 1.8, 95% CI 1.0–3.2). Higher lutein intake tended to reduce the risk of AMD, with OR 0.6 (95% CI 0.4–1.0) for the third tertile versus the first tertile. Additional adjustment for the other seven genetic loci (multivariate model 2) did not alter these associations. There were no substantial differences between GA and NV in the analyses of these covariates.

We assessed the effect of interactions between *LIPC* genotypes and lifestyle factors on risk of AMD; results are shown in Table 4. There were no statistically significant interactions, meaning that the effect of the gene did not vary significantly according to a specific category of the behavioral factor. Higher BMI and cigarette smoking tended to increase risk of AMD in the CC and CT genotype groups; numbers were too small in the TT group to identify BMI and smoking effects for this genetic subgroup.

**Table 4:** Assessment of effect of interactions between hepatic lipase-C (*LIPC*) genotype and lifestyle factors on risk of age-related macular degeneration (AMD).

** **	** **	***LIPC* genotype**			**P (Trend) for Number of T alleles**
** **	** **	**CC**	**CT**	**TT**	** **
Number of cases					
	Combined Advanced AMD	293	224	28	
	Geographic Atrophy	82	50	7	
	Neovascular AMD	211	174	21	
Number of controls		128	121	26	
		OR (CI)*	OR (CI)*	OR (CI)*	
**BMI**	Combined Advanced AMD				
	<25	1.0	1.1 (0.6 - 2.0)	0.6 (0.2 - 1.8)	
	25+	1.7 (1.0 - 2.8)	1.4 (0.8 - 2.3)	0.7 (0.3 - 1.6)	
	P (Interaction)		0.40 (CT vs. CC)	0.59 (TT vs. CC)	0.4
**Smoking**	Combined Advanced AMD				
	Never	1.0	1.1 (0.7 - 1.8)	0.6 (0.2 - 1.3)	
	Ever	2.1 (1.4 - 3.3)	1.6 (1.0 - 2.5)	0.7 (0.3 - 1.8)	
	P (Interaction)		0.27 (CT vs CC)	0.43 (TT vs. CC)	0.3
**Lutein: **	Combined Advanced AMD				
calorie adjusted,	≤mean†	1.0	0.8 (0.5 - 1.3)	0.7 (0.3 - 1.7)	
sex specific	> mean†	0.7 (0.4 - 1.1)	0.7 (0.4 - 1.1)	0.2 (0.1 - 0.5)	
	P (Interaction)		0.62 (CT vs. CC)	0.11 (TT vs. CC)	0.83

*OR=Odds Ratio (adjusted for age, gender, education and all other variables in the table), CI=95% Confidence Interval †Mean Lutein=1355 µg.

Shown in Table 5 are the effects of interactions between *LIPC* genotypes and other genes on risk of advanced AMD. There was a borderline significant interaction between *LIPC* and the *CFI* rs10033900 and *CFH* rs1410996 genotypes. *LIPC* appears to be more protective when *CFI* rs10033900 is CC or CT as opposed to TT. *LIPC* is more protective when *CFH* rs1410996 is CT or TT versus CC.

**Table 5:** Assessment of effect of interactions between hepatic lipase (*LIPC*) genotype (rs10468017) and other genes on risk of age-related macular degeneration.

		**CC**	***LIPC* genotype**
**Number of cases**	** **	** **	**CT & TT**
	Combined Advanced AMD	293	252
	Geographic Atrophy	82	57
	Neovascular AMD	211	195
Number of controls		128	147
		OR (CI) *	OR (CI) *
*CFH*: rs1061170 (Y402H)	Combined Advanced AMD		
	TT	1.0	0.5 (0.3 - 0.9)
	CT	1.7 (1.0 - 2.9)	1.9 (1.1 - 3.3)
	CC	5.6 (2.8 - 11.0)	5.3 (2.7 - 10.3)
	p (interaction)		0.12 (CT-TT versus CC)
*CFH*: rs1410996	Combined advanced AMD		
	TT	1.0	0.6 (0.2 - 2.0)
	CT	2.8 (1.1 - 6.7)	1.6 (0.7 - 4.0)
	CC	7.8 (3.2 - 19.0)	9.9 (4.0 - 24.5)
	p (interaction)		0.05 (CT -TT vs CC)
*ARMS2*/*HTRA1*: rs10490924	Combined advanced AMD		
	GG	1.0	0.9 (0.6 - 1.5)
	GT	3.9 (2.4 - 6.3)	2.6 (1.6 - 4.1)
	TT	21.2 (6.3 - 71.9)	12.8 (4.3 - 37.9)
	p (interaction)		0.32(CT-TT versus CC)
*CFB*: rs641153 (R32Q)	Combined advanced AMD		
	CC	1.0	0.9 (0.6 - 1.3)
	CT/TT	0.3 (0.2 - 0.6)	0.1 (0.1 - 0.3)
	p (interaction)		0.14 (CT-TT versus CC)
*C2*: rs9332739 (E318D)	Combined advanced AMD	1.0	
	GG	0.4 (0.1 - 1.4)	0.9 (0.6 - 1.2)
	CG/CC		0.2 (0.1 - 0.5)
	P (Interaction)		0.13 (CT-TT versus CC)
*C3*: rs2230199 (R102H)	Combined Advanced AMD		1.0 (0.6 - 1.4)
	CC	1.0	
	CG	1.8 (1.1 - 2.8)	1.4 (0.9 - 2.2)
	GG	8.8 (2.0 - 39.4)	1.5 (0.6 - 3.8)
	p (interaction)		0.10 (CT-TT versus CC)
*CFI*: rs10033900	Combined advanced AMD		
	CC	1.0	0.7 (0.4 - 1.3)
	CT	1.5 (0.9 - 2.5)	0.9 (0.5 - 1.5)
	TT	1.1 (0.6 - 2.0)	2.1 (1.1 - 4.1)
	p (interaction)		0.04 (CT-TT versus CC)

*OR=Odds Ratio (adjusted for age, gender, education, smoking, BMI, calorie adjusted lutein, and treatment), CI=95% Confidence Interval.

## Discussion

To our knowledge, this is the first evaluation of the relationship between the *LIPC* functional variant and advanced AMD while controlling for demographic and behavioral factors including BMI, smoking, and dietary factors, as well as previously identified AMD genes. *LIPC* and environmental factors were independently associated with advanced AMD, the leading cause of visual impairment and vision-related reduced quality of life among elderly individuals. Controlling for the *LIPC* genotype, modifiable lifestyle factors, including higher BMI, smoking, and lower dietary lutein, were significantly associated with increased risk of advanced AMD. Similar to our previous findings with other genetic variants [38–41], there was an independent effect of both the genetic and modifiable behavioral factors when they were considered simultaneously, but there were no significant interactions between the genetic and environmental factors on risk of AMD. There was a possible gene-gene association, however, between *LIPC* and *CFH* rs1410996, and a possible interaction effect between *LIPC* and both *CFH* rs1410996 and *CFI* rs10033900 variants in terms of risk of AMD, but no other associations or interactions were seen between *LIPC* and the other known AMD genes.

The association between *LIPC* polymorphisms and AMD is biologically plausible because this gene is involved with the HDL cholesterol pathway, and cardiovascular disease (CVD) risk factors are associated with AMD [42]. It has been suggested that CVD could also be a model for the role of cholesterol in AMD [35]. Modifiable factors for CVD such as smoking and BMI are associated with both cholesterol [43,44] and AMD. High BMI and smoking are associated with increased LDL and lower HDL [43,44]. In a separate report, we evaluated the relationship between serum lipids, *LIPC* and AMD, and found an inverse (protective) association between HDL and AMD, and a positive (adverse) association with higher LDL and total cholesterol [36]. When we evaluated both *LIPC* and HDL together, the level of serum lipid did not appear to modify the effect of *LIPC* on AMD [36], suggesting that although *LIPC* regulates level of HDL, this may not be the direct mechanism whereby *LIPC* reduces risk of AMD. HDL transports lutein and zeaxanthin and these carotenoids are also associated with reduced risk of AMD [7–10,34–36]. A change in the efficiency of carotenoid delivery is one mechanism by which *LIPC* genetic variation could be related to AMD [29].  Further research into the mechanisms of *LIPC* and the HDL pathway in the pathogenesis of AMD are needed.

Strengths of the study include the large, well characterized population of patients with and without advanced AMD from various geographic regions around the US, the standardized collection of risk factor information, direct measurements of height and weight, and classification of maculopathy by ophthalmologic examinations and fundus photography. Misclassification was unlikely, since grades were assigned without knowledge of risk factors or genotype. We controlled for known AMD risk factors, including age and education, as well as antioxidant status, in the assessment of BMI, smoking, dietary lutein, and genotype. The environmental and genetic risk factors were independently associated with AMD, when considered simultaneously. There may be some other unmeasured factors that might still be confounding these relationships, but they would have to be highly related to genotype, smoking and BMI, and a strong risk factor for AMD to explain these results. Although this is a selected population, cases likely represent the typical patient with AMD seen in clinical setting. The overall population is similar to others in this age range in terms of smoking and prevalence of obesity, as well as the distribution of the *LIPC* genotype. Furthermore, the biologic effects of *LIPC* and the modifiable factors are not likely to differ in major ways among various European populations with AMD. This study of moderate sample size may not have sufficient power to detect small to intermediate interaction effects between genes or between genes and environmental factors. Larger studies, as well as prospective studies, are needed to confirm and expand upon these findings.

### Conclusion

*LIPC* is independently associated with reduced risk of advanced AMD, adjusting for demographic and environmental variables. Both genetic susceptibility and behavioral and lifestyle factors modify risk of developing AMD.
